# Acoustic Stimulation Improves Memory and Reverses the Contribution of Chronic Sleep Deprivation to Pathology in 3xTgAD Mice

**DOI:** 10.3390/brainsci12111509

**Published:** 2022-11-06

**Authors:** Shunjie Liu, Qingfeng Lei, Yunyun Liu, Xiaofeng Zhang, Zhong Li

**Affiliations:** 1 Department of Neurology, The Sixth Affiliated Hospital, Sun Yat-sen University, Guangzhou 510655, China; 2 Shenzhen Research Institute, Sun Yat-sen University, Shenzhen 518000, China; 3 Guangdong Provincial Key Laboratory of Brain Function and Disease, Guangzhou 510080, China

**Keywords:** Alzheimer’s disease, acoustic stimulation, sleep deprivation, spatial memory

## Abstract

**Objective**: Acoustic stimulation during sleep is believed to enhance slow waves, which are critical to memory consolidation. However, clinical trials of acoustic stimulation have yielded mixed results concerning its effectiveness in improving human memory. A few studies have implied that acoustic stimulation ameliorates the pathology of Alzheimer’s disease (AD) in mice with normal sleep. Here, we explored the effect of acoustic stimulation on 3xTgAD mice suffering from chronic sleep deprivation, as these data may shed light on the potential use of acoustic stimulation in AD patients with insomnia. **Methods**: Twenty-four 8-month-old 3xTgAD mice were randomly and equally divided into three groups: the normal sleep group (S group), the sleep deprivation group (SD group), and the acoustic stimulation group (AS group). During a 14-day sleep intervention, the SD and AS groups received 6 h of sleep deprivation per day, and the AS group also received acoustic stimulation in the dark phase. Then, the mice underwent Morris water maze (MWM) tests and arterial spin labelling (ASL) magnetic resonance imaging (MRI) scans and were sacrificed for pathological evaluation. **Results**: The three groups showed similar stress levels. The S and AS groups exhibited better spatial memory, better brain perfusion, and milder amyloid β (Aβ) and tau pathology than the SD group, although no significant discrepancies were found between the S and AS groups. **Conclusions**: Acoustic stimulation may exert a protective effect in 3xTgAD mice by improving spatial memory, enhancing the blood supply of the brain, and reversing the contribution of chronic sleep deprivation to Aβ and tau pathology to mimic the effect of normal sleep patterns.

## 1. Introduction

With the rapidly increasing population of elderly people above 65 years of age, Alzheimer’s disease (AD), which is considered the most common type of dementia among elderly people [1], has become a threat to public health worldwide [2]. AD is characterized by amyloid β (Aβ) plaque accumulation and tau protein hyperphosphorylation in the brain; its main symptoms are obvious impairments in memory and other cognitive domains [3]. Over the last few decades, scientists have made great efforts to develop drugs in order to cure AD. Regretfully, most of these drugs have failed in clinical trials because of unsatisfactory effectiveness or serious side effects [4]. The failure to develop a pharmacological cure has encouraged scientists to investigate non-drug therapies.

Sleep plays an indispensable role in maintaining normal functions of our body [5], and sleep disturbance is a physiological hallmark of patients with AD [6]. This is why a great proportion of non-drug therapies are aimed at sleep intervention [7].

Sleep can generally be divided into rapid eye movement (REM) sleep and non-rapid eye movement (NREM) sleep [8]. Slow waves whose frequency is less than 1 Hz are prominent hallmarks of NREM sleep [8]. Evidence suggests that slow waves are critical to memory consolidation [9], since they may promote the transformation of memory from a volatile and hippocampus-dependent state to a stable and increasingly hippocampus-independent state [10,11]. Therefore, approaches to slow-wave enhancement, such as acoustic stimulation, have been developed to improve memory ability [12,13,14].

However, although previous studies have found that acoustic stimulation may indeed entrain and enhance slow waves during sleep, its effect on memory consolidation is still uncertain and controversial [15]. On the one hand, while several clinical studies showed that acoustic stimulation might help preserve memory function in young, middle-aged and elderly adults [16,17], other clinical studies concluded that acoustic stimulation did not produce significant effects on human memory [18,19]. On the other hand, a few animal studies have investigated the relationships between specific frequencies of acoustic or light stimulation and AD, providing convincing evidence that these stimuli may ameliorate AD-related pathology and improve the cognitive abilities of mice [20,21]. However, these animal studies explored the conditions of AD mouse models with a normal sleep-wake cycle instead of mice suffering from chronic sleep deprivation; the latter is a more accurate model of the large proportion of AD patients who suffer from long-term insomnia in real life.

Therefore, on the ground of the findings and downsides of previous studies, we designed this study. In this study, we imposed chronic sleep deprivation on 3xTgAD mice, simulating the conditions of numerous AD patients who suffer from sleep disorders, and we investigated the effect of suitable acoustic stimuli on AD pathology and memory. The findings of our study may elucidate the role of acoustic stimulation in curing AD, thereby providing theoretical support for the use of related devices in clinical practice.

## 2. Materials and Methods

### 2.1. Animals

This study used 3xTgAD mice (12 males and 12 females), all of which were 8 months old at the start of the experiment. Mice of that line were acquired from Ailing Fei Bioengineering Co., Ltd. (Jiangsu, China), and they were bred in the animal room of the Sixth Affiliated Hospital, Sun Yat-sen University; the experimental animals were selected among their progeny, which were reared in the same facility. The animal room was operated on a universal 12 h light: 12 h dark cycle (light on [zeitgeber time (ZT) 0] at 7:00 A.M.) at a constant indoor temperature of 23 ± 1 °C. Water and food were available ad libitum and replaced every morning. The Institutional Animal Care and Use Committee of the Sixth Affiliated Hospital of Sun Yat-sen University set restrictions for the experiments and approved the mouse care protocols according to the National Institutes of Health guidelines.

### 2.2. Study Design

**Figure 1:**
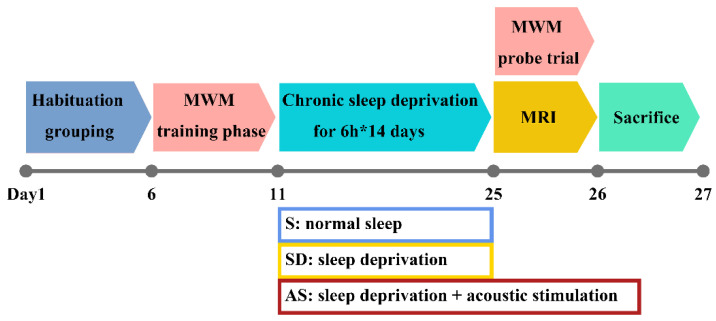
Timeline of experimental design. After habituation for 5 days and performing MWM training for 5 days, mice received 14-day sleep deprivation and acoustic stimulation according to grouping. Then, all groups underwent MWM probe trial and MRI scans. Following these examinations, they were sacrificed for pathology evaluation.

Mice were equally and randomly divided into the following three groups, each of which consisted of 8 mice (4 males and 4 females): (a) the normal sleep (S) group; (b) the sleep deprivation group, called the SD group; (c) and the acoustic stimulation group, called the AS group. Given the aggressive nature of mature 3xTgAD mice, mice in the same group were accommodated in a large home cage with wire nets dividing the cage into 8 separate rooms, which ensured that each mouse had enough space for physical activity but could not attack the other mice. By applying this setting, we could prevent fights among mice while still avoiding the effect of social isolation on their behaviour to some extent. Before the experiments, the mice were given 5 days to adjust to their environment. Next, the mice underwent Morris water maze (MWM) training and sleep deprivation. Then, the mice underwent brain magnetic resonance imaging (MRI) and, following, MWM probe trials (these two tests were conducted in the morning on the 25th day); finally, they were sacrificed under deep anaesthesia for hippocampal tissue collection (Figure 1).

### 2.3. Sleep Deprivation and Acoustic Stimulation

The animal rooms were lined with sound-proof foam to block out noise, and each group was housed in a separate room. During the 14-day sleep intervention period, the S group was able to sleep ad libitum, while the SD and AS groups received 6 h of sleep deprivation from ZT 0 to ZT 6 each day. To deprive the mice of sleep without causing stress responses, gentle handling was applied by technicians to wake the mice, which expressed behavioural signs of sleepiness according to visual observation, as described in a previous study [22]. In the room accommodating the AS group, a Bluetooth speaker (Philips; China) was placed above the cage, beyond the reach of the mice, to produce continuous tones during the dark phase (ZT 12 to ZT 24), because mice suffering from sleep deprivation during the light phase are believed to exhibit recovery sleep during the dark phase [23,24]. These continuous tones were set to a frequency of 40 Hz, a duration of 50 ms and a volume of 60 dB, with a 1000 ms interstimulus interval; these parameters are based on previous studies with slight modification [14,20].

To ensure that the stress levels of the animals did not increase rapidly during the deprivation period, plasma cortisol concentrations and body weight were measured; additionally, skin lesions were observed before deprivation, in the middle of deprivation (the 7th day of the sleep deprivation period) and immediately after deprivation.

The skin lesions of interest were erythematous papules (small, raised and inflamed regions) on the paws or reddish-brown circles under the epidermis of the tail [25]. Body weight was measured at ZT 0 at these three time points according to a previous protocol [24].

For plasma cortisol measurement, venous blood (approximately 0.1 mL) was obtained from the vena caudalis of the mice through a capillary tube (Sutter; Sacramento, CA, USA) at ZT 0 at these three time points to evade the effects of circadian rhythm on plasma cortisol concentrations. Next, the blood samples were transferred to polypropylene tubes (Jet Bioful; Guangzhou, China) and centrifuged (2000× *g*, +4 °C, 10 min). The cortisol levels in the supernatants were measured using commercial enzyme-linked immunosorbent assay (ELISA) kits (Cusabio; Houston, TX, USA). The measurement procedures were performed on the basis of the manufacturer’s instructions. Each sample was analysed in triplicate, and the mean value was determined as the final result. We also conducted vaginal smears from the female mice and visually observed the appearance of the vagina to track the oestrous cycle. We found that none of the female animals were in the oestrus period during the sleep deprivation period; therefore, we might exclude the influences of the oestrous cycle on plasma cortisol concentrations.

### 2.4. MWM Experiment

The MWM experiment was applied to measure spatial memory. The training phase and probe trial were conducted before and after chronic SD, respectively, to evaluate the retention of spatial memory in mice.

We conducted the MWM test according to previous protocols [26]. The MWM apparatus included a round pool of water (a diameter of 122 centimetres, the wall was painted white) and a hidden platform (a diameter of 10 centimetres, approximately 1 centimetre under the water’s surface). During the training phase, the animals were released 4 times on each day from different starting points for 5 consecutive days to practice locating the platform. During the probe trial, the hidden platform was removed, and the mice freely explored the pool for 60 s. The data were recorded and analysed with a video-tracking system (ANY-maze 7.0, Stoelting; Wood Dale, IL, USA).

### 2.5. Cerebral Blood Flow (CBF) Measurement

CBF levels were measured by arterial spin labelling (ASL) MRI. MRI scans were conducted using a 9.4-T MRI scanner (Bruker; Bremen, Germany) with a self-shielded gradient system (12 cm in diameter) and a 23-mm surface coil. The system was connected to Topspin version 2.1 and Paravision version 5.1 software (Bruker; Bremen, Germany). The procedures for the MRI tests and the parameters of the MRI system were set according to the instructions of the machine. Briefly, mice were anaesthetized with inhaled isoflurane (4% for induction and 1.5% for maintenance). Heart rate, respiratory rate as well as body temperature were continuously monitored. T2-weighted images were obtained by using rapid acquisition with relaxation enhancement (RARE) with the following parameters: RARE factor, 8; field of view (FOV), 20 × 20 mm; repetition time (TR)/echo time (TE), 10104.89/42.0 ms; matrix size, 256 × 256; slice thickness, 0.5 mm; the number of slices, 15. ASL images were obtained from the T2-weighted images and then reconstructed using Paravision version 5.1 software. Within the ASL images, we selected 8 regions of interest (ROIs), i.e., the bilateral cortex, hippocampus, thalamus, and amygdala, for CBF measurement.

### 2.6. Hippocampal Tissue Preparation

On day 26, anaesthetized mice were perfused with 4% paraformaldehyde (PFA) (Servicebio; China) and phosphate-buffered saline (PBS) (Servicebio; Wuhan, China). Then, death was confirmed by cervical dislocation, and the hippocampal tissues were carefully removed. Tissues were incubated in 4% PFA at +4 °C overnight for fixation before being placed in PBS with 30% sucrose for 48 h for dehydration. Two 20-μm-thick coronal sections (300 μm interval) of the dentate gyrus area were acquired from each hippocampus by using a freezing microtome (Leica; Germany). The remaining part of each hippocampus was stored at −80 °C until use.

### 2.7. Immunofluorescence and Plaque Analysis

Slices of the dentate gyrus area were incubated in blocking buffer with 3% bovine serum albumin (BSA) at room temperature for 30 min. Slices were incubated with primary antibodies at +4 °C overnight and then incubated with suitable fluorophore-conjugated secondary antibodies at room temperature for 1 h in the dark. The nuclei were dyed with 4,6-diamidino-2-phenylindole (DAPI, 1:5000, Abcam #ab104139; Cambridge, UK). The primary antibodies used were anti-Aβ (1:200, Invitrogen #MA1-25493; Waltham, MA, USA) to recognize Aβ plaques and anti-GFAP (1:1000, Invitrogen #PA1-10004; Waltham, MA, USA) to recognize astrocytes. Alexa Fluor–conjugated secondary antibodies (1:500, Abcam; Cambridge, UK) were used. Further analyses, including quantification and analysis of Aβ plaques, were performed on the slice with the maximum signal intensity from each mouse. ImageJ software (National Institutes of Health; Bethesda, Rockville, MD, USA) was applied to measure the diameter of each plaque. Then, plaques were classified into four quartiles based on their diameter, and the number of plaques within each quartile was divided by the total number of plaques to acquire an index for the size of plaques.

### 2.8. ELISAs

ELISAs were used to measure Aβ levels in hippocampal tissues. After sections were used for immunostaining, the remainder of each hippocampus was homogenized in PBS with protease and phosphatase inhibitor cocktails (Servicebio; Wuhan, China) before being centrifuged (12,000× *g*, +4 °C, 20 min). The supernatant was collected, and ELISAs for soluble Aβ_1–40_ and Aβ_1–42_ were performed.

Insoluble Aβ levels were measured, as described in a previous study [27]. The insoluble contents were mechanically homogenized in 5 M guanidine and centrifuged (12,000× *g*, +4 °C, 20 min). Next, the supernatant was collected, and ELISAs for insoluble Aβ_1–40_ and Aβ_1–42_ were performed. The Aβ_1–40_ and Aβ_1–42_ kits applied were #KMB3481 and #KMB3441 (Invitrogen; Carlsbad, CA, USA).

### 2.9. Western Blot Analysis

Western blot analyses were conducted on the collected supernatant mentioned above. First, the total protein concentration in every sample was measured by using a bicinchoninic acid (BCA) protein assay kit (Abcam; Cambridge, UK). Next, proteins were separated by 10% SDS–PAGE and transferred to commercial polyvinylidene difluoride (PVDF) membranes (Abcam; Cambridge, UK). We blocked the membranes in TBST with 5% non-fat milk at room temperature for 1 h. Subsequently, membranes were incubated with specific anti-tau antibodies at +4 °C for one night before being incubated with HRP-conjugated secondary antibodies at room temperature for one hour. The tau antibodies used were anti-tau-pThr 181 (1:1000, Invitrogen #701530; Carlsbad, CA, USA), anti-tau-pThr 231 antibody (1:1000, Invitrogen #44-746G; Carlsbad, CA, USA) and anti-tau antibody (1:1000, Invitrogen #AHB0042; Carlsbad, CA, USA). An Image Quant LAS 4000 imager (GE Healthcare Bio-Sciences; Uppsala, Sweden) and ImageJ software (National Institutes of Health; Bethesda, MD, USA) were applied for the detection and quantification of protein expression. The expression levels of tau proteins were normalized to those of β-actin.

### 2.10. Statistical Analysis

The normality of the data in our study was examined through the Shapiro–Wilk test. Group differences in normally distributed data with homogeneous variance were analysed through one-way analysis of variance (ANOVA) followed by post hoc comparisons using Fisher’s least significant difference (LSD) test. Otherwise, group differences in non-normally distributed data were analysed using the Kruskal–Wallis test followed by the Mann–Whitney *U* test, and the Bonferroni correction was applied to the *p* values. Because the proportion of Aβ plaques in each quartile is an ordinal categorical variable, it was compared by using the Kruskal–Wallis test followed by post hoc comparisons using the Mann–Whitney U test with Bonferroni-corrected *p* values. The 25.0 IBM SPSS (IBM Corp.; Armonk, NY, USA) was used for statistical analysis. Data are expressed as the mean ± S.E.M. In the present study, statistical significance was defined by *p* < 0.05.

## 3. Results

### 3.1. Chronic Sleep Deprivation Did Not Cause Stress Responses

In this study, we observed skin lesions and measured body weight and plasma cortisol as indicators of stress responses. No significant differences in skin lesions on the surface of the paws or tails during sleep deprivation were observed among the three groups (Table 1).

**Figure 2:**
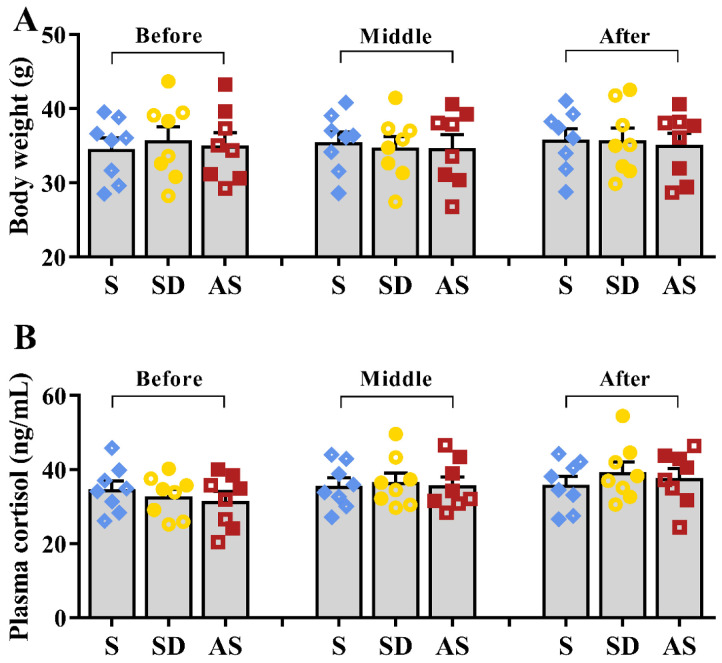
Chronic sleep deprivation did not cause stress responses. (**A**) Body weight during the sleep deprivation period. (**B**) Plasma cortisol levels during the sleep deprivation period. One-way ANOVA followed by two-sample LSD *t* tests is used. Data are expressed as mean ± SEM. Closed dots constitute male mice, and open dots constitute female mice.

Similarly, no significant differences in body weight or plasma cortisol levels were found among the three groups (Figure 2A,B). There were no significant sex differences in any groups. These results suggest that the three groups exhibited similar stress levels during sleep deprivation, meaning that chronic sleep deprivation by gentle handling did not lead to serious stress responses.

### 3.2. Acoustic Stimulation Improves Spatial Memory

**Figure 3:**
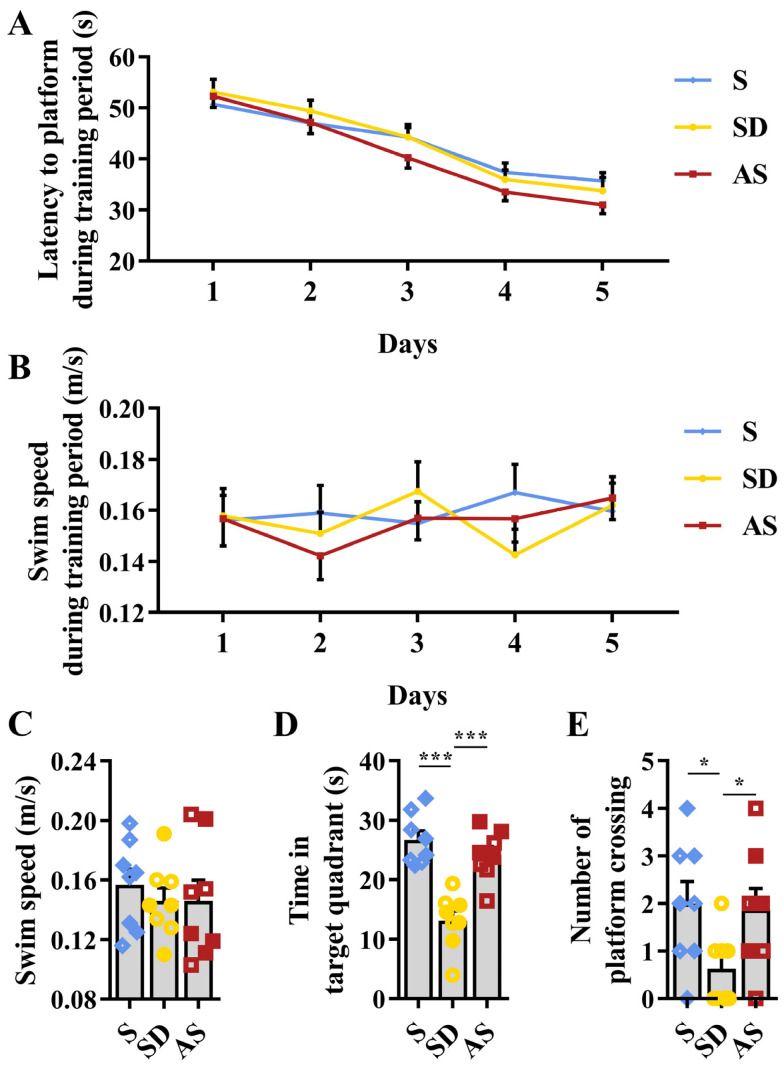
Acoustic stimulation improved spatial memory. (**A**) Latency to reach a hidden platform during the 5-day training period. (**B**) Swim speed during the 5-day training period. (**C**) Swim speed in the probe trial. (**D**) Time spent in the target quadrant in the probe trial. (**E**) The number of crossings of the original platform-site in the probe trial. One-way ANOVA followed by two-sample LSD *t* tests is used. Data are expressed as mean ± SEM. Closed dots constitute male mice, and open dots constitute female mice. * *p* < 0.05, *** *p* < 0.001.

The MWM experiment was applied to test the spatial memory of 3xTgAD mice. Before sleep deprivation, no differences in latency to reach the platform or in swimming speed were found among the three groups (Figure 3A,B). In the probe trial after sleep deprivation, while no discrepancies in swimming speed were found among the three groups (Figure 3C), we observed significant discrepancies in the time spent in the target quadrant (*F*_2,21_ = 21.838, *p* < 0.001) and in the number of transits across the former platform location (*F*_2,21_ = 3.631, *p* = 0.044) among the three groups. Then, post hoc tests revealed that the SD group stayed less in the target quadrant than the S and AS groups, while we did not find significant discrepancies between the S and AS groups (Figure 3D); the SD group crossed the original platform position fewer times than the S and AS groups, while we did not find significant discrepancies between the S and AS groups (Figure 3E). There were no significant sex differences in any groups. The MWM results suggested that acoustic stimulation improved spatial memory deteriorated by chronic sleep deprivation.

### 3.3. Acoustic Stimulation Increases Regional CBF

**Figure 4:**
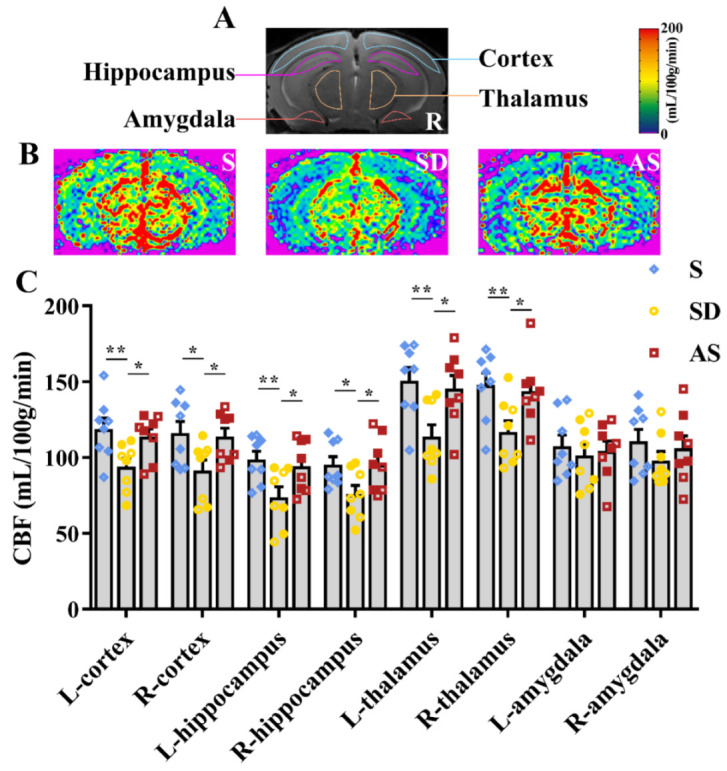
Acoustic stimulation increased regional CBF. (**A**) A total of 8 ROIs (bilateral cortex, hippocampus, thalamus and amygdala) for regional CBF measurement. (**B**) Representative CBF images. Red indicates high CBF, and blue indicates low CBF. (**C**), Quantitative analysis. One-way ANOVA followed by two-sample LSD *t* tests is used. Data are expressed as mean ± SEM. Closed dots constitute male mice, and open dots constitute female mice. * *p* < 0.05, ** *p* < 0.01.

For each mouse that underwent MRI scanning, a T2-weighted slice of 0.5 mm thickness was examined, and the bilateral cortex, hippocampus, thalamus, and amygdala were identified in the slice (Figure 4A). These 8 ROIs were transferred from the T2-weighted images to the corresponding CBF maps, and the average CBF values were collected and calculated from the ROIs.

We found differences in regional CBF values in the left cortex (*F*_2,21_ = 4.816, *p* = 0.019), right cortex (*F*_2,21_ = 4.006, *p* = 0.034), left hippocampus (*F*_2,21_ = 4.694, *p* = 0.021), right hippocampus (*F*_2,21_ = 3.794, *p* = 0.039), left thalamus (*F*_2,21_ = 5.823, *p* = 0.010) and right thalamus (*F*_2,21_ = 4.695, *p* = 0.021). Post hoc tests suggested that the SD group showed lower regional CBF values in these six ROIs than the S and AS groups, while we did not find significant discrepancies between the S and AS groups. Moreover, no differences in regional CBF values of the bilateral amygdala were found among the three groups (Figure 4B,C). There were no significant sex differences in any groups. The MRI results suggested that acoustic stimulation increased CBF in brain areas associated with memory and behaviour, counteracting the effect of chronic sleep deprivation.

### 3.4. Acoustic Stimulation Reverses Aβ Pathology

**Figure 5:**
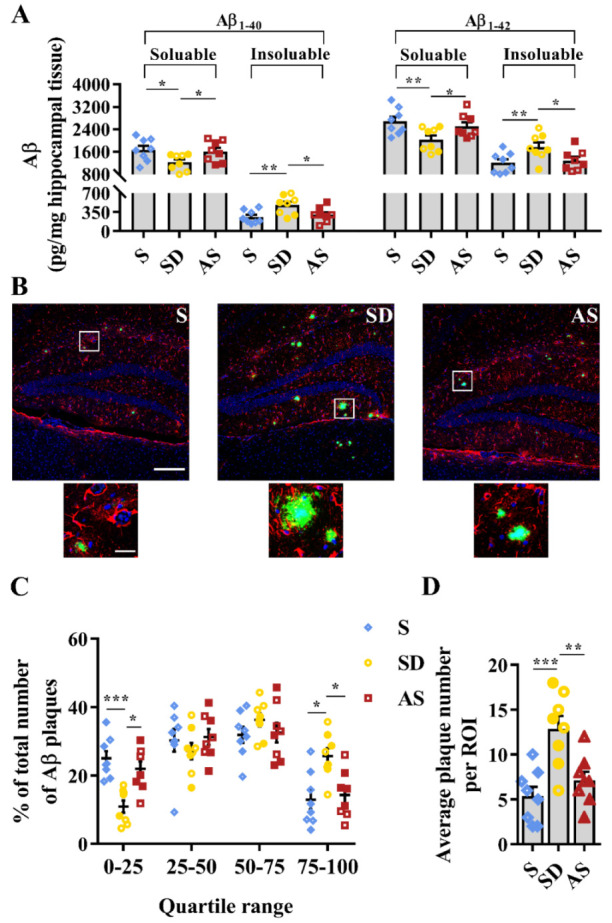
Acoustic stimulation reversed Aβ pathology. (**A**), Soluble and insoluble Aβ_1–40_ and Aβ_1–42_ levels in hippocampal tissue. (**B**) Representative immunofluorescence images of astrocytes (red), Aβ plaques (green) and nuclei (blue) in the dentate gyrus area. Scale bar, 200 µm; scale bar of insets, 20 µm. (**C**) Aβ plaques were ranked and divided into quartiles according to their diameter (the Kruskal–Wallis test followed by the Mann–Whitney U test with Bonferroni-corrected *p* values). (**D**) Quantitative analysis of the number of Aβ plaques. One-way ANOVA followed by two-sample LSD *t* tests is used. Data are expressed as mean ± SEM. Closed dots constitute male mice, and open dots constitute female mice. * *p* < 0.05, ** *p* < 0.01, *** *p* < 0.001.

Commercial ELISA kits were used to measure Aβ levels in hippocampal tissues. There were significant differences in the levels of soluble Aβ_1–40_ (*F*_2,21_ = 3.560, *p* = 0.047), insoluble Aβ_1–40_ (*F*_2,21_ = 5.397, *p* = 0.013), soluble Aβ_1–42_ (*F*_2,21_ = 4.704, *p* = 0.021), and insoluble Aβ_1–42_ (*F*_2,21_ = 4.636, *p* = 0.022) among the three groups. Then, post hoc tests revealed that the SD group showed lower soluble Aβ_1–40_ and soluble Aβ_1–42_ levels as well as higher insoluble Aβ_1–40_ and insoluble Aβ_1–42_ levels than the S and AS groups, while we did not find significant discrepancies between the S and AS groups (Figure 5A).

Furthermore, we investigated the condition of Aβ plaques by confocal laser scanning microscopy, observing that the SD group showed more and larger Aβ plaques in the dentate gyrus than the S and AS groups (Figure 5B). We divided plaques into quartiles based on diameter, finding significant differences in the proportions of plaques within the first quartile (*H* = 13.715, *p* = 0.001) and the fourth quartile (*H* = 9.500, *p* = 0.009). Post hoc tests indicated a lower proportion of plaques in the first quartile and a higher proportion of plaques in the fourth quartile in the SD group than in the S and AS groups, while there were no differences between the S and AS groups (Figure 5C). Quantitative analysis suggested that there were significant differences in average plaque count per ROI in the dentate gyrus among the three groups (*F*_2,21_ = 11.464, *p* < 0.001), and post hoc tests indicated that the SD group averaged more plaques per ROI than the S and AS groups, while we did not find significant discrepancies between the S and AS groups (Figure 5D). There were no significant sex differences in any groups.

### 3.5. Acoustic Stimulation Reduces Tau Proteins

**Figure 6:**
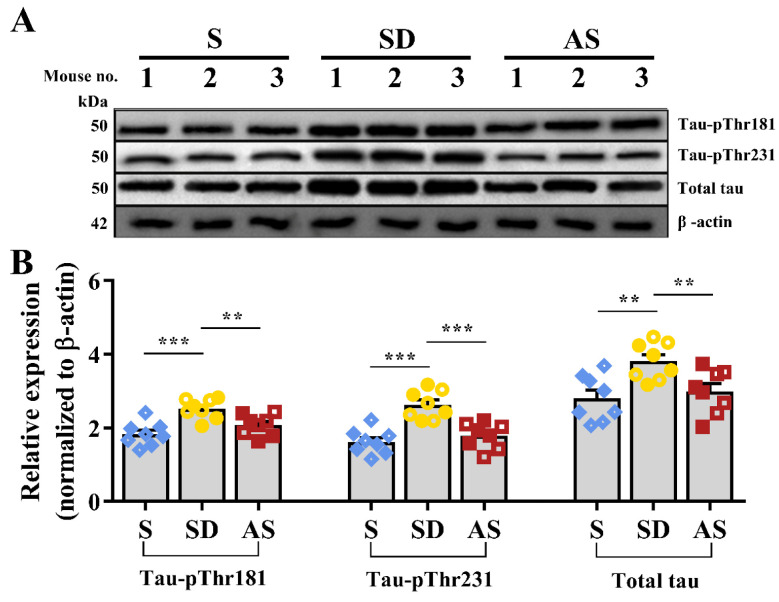
Acoustic stimulation inhibited tau pathology. (**A**) Representative images of Western blot. (**B**) The quantitative values of tau proteins. One-way ANOVA followed by two-sample LSD *t* tests is used. Data are expressed as mean ± SEM. Closed dots constitute male mice, and open dots constitute female mice. ** *p* < 0.01, *** *p* < 0.001.

Western blotting was applied to measure tau protein expression (Figure 6A). Quantitative analysis suggested significant differences in tau-pThr 181 (*F*_2,21_ = 11.674, *p* < 0.001), tau-pThr 231 (*F*_2,21_ = 18.170, *p* < 0.001) and total tau (*F*_2,21_ = 6.989, *p* = 0.005), and post hoc tests indicated that the SD group showed higher expression of these three tau proteins than the S and AS groups, while we did not find significant discrepancies between the S and AS groups (Figure 6B). There were no significant sex differences in any groups.

## 4. Discussion

To our knowledge, we are the first to explore the effect of acoustic stimulation on AD mouse models subjected to chronic sleep deprivation. We found that suitable acoustic stimuli improved spatial memory, increased regional CBF in critical brain areas and reversed Aβ and tau pathology that had been exacerbated by chronic sleep deprivation in AD mouse models.

One major challenge of our experiment was determining the parameters of AS. Considering the discrepancies between mice and humans, it would be unwise to adopt exactly the same acoustic parameters applied in clinical trials. Therefore, regarding the frequency and loudness of acoustic stimuli, we applied the same parameters (frequency at 40 Hz and loudness at 60 dB) used in a previous study focusing on a 5xFAD mouse model [20]. Then, to investigate the effectiveness of the most widely used and available machines in clinical practice, we adopted the duration and interstimulus interval parameters of acoustic stimuli applied in clinical trials (50 ms duration and 1000 ms interstimulus interval) [14]. Regarding the timing of acoustic stimuli, we did not apply them for the whole day, as in a previous study [20]; instead, we applied acoustic stimulation only in the dark phase. We made this choice for two reasons: (a) the potential mechanism of acoustic stimulation is that it may entrain and enhance slow waves during sleep; (b) the mice in the AS group were subjected to sleep deprivation during the light phase, which means that most of their sleep might occur during the dark phase. In other words, the acoustic stimulation was timed in this manner so that it would largely overlap with sleep.

In this study, we explored the effect of acoustic stimulation during probable sleep in the following aspects.

First, we investigated the effect of acoustic stimulation on memory. Memory deficits are regarded as the most prominent cognitive impairment associated with AD [28]. In the present study, we separated the MWM test into two parts, namely, a training phase before chronic sleep deprivation and a probe trial after chronic sleep deprivation, to evaluate the long-term memory retention of mice. We found that spatial memory declined significantly because of chronic sleep deprivation (based on a comparison between the S and SD groups), and acoustic stimulation significantly improved spatial memory (based on a comparison between the AS and SD groups). These results indicate that acoustic stimulation could reduce the ill effects of chronic sleep deprivation on long-term memory.

Second, we examined the effect of AS on brain blood supply. It is widely recognized that disturbance of brain blood supply, characterized by regional hypoperfusion and vascular dysfunction [29], is a persistent symptom of AD and is positively associated with the development of this disease [30]. In the present study, we measured regional CBF by ASL MRI to assess the severity of hypoperfusion and vascular dysfunction. We found that CBF in the bilateral cortex, hippocampus, and thalamus decreased significantly as a result of chronic SD (based on a comparison between the S and SD groups), and acoustic stimulation significantly increased CBF in these regions (based on a comparison between the AS and SD groups). These results indicate that acoustic stimulation could counteract the contribution of chronic sleep deprivation to the disturbance of the blood supply in critical brain areas. Interestingly, we also found that CBF in the bilateral amygdala remained more or less unchanged after sleep deprivation or acoustic stimulation; this observation should be investigated further in future studies because the underlying mechanism is still unknown.

Third, we examined the effect of AS on Aβ pathology and tau protein levels. Aβ plaque deposition and tau hyperphosphorylation are two prominent pathological hallmarks of AD [31]. In this study, we found that chronic SD fuelled Aβ plaque deposition and tau hyperphosphorylation (based on a comparison between the S and SD groups), and acoustic stimulation reversed these pathological changes (based on a comparison between the AS and SD groups). Regarding the potential mechanisms, we speculate that acoustic stimulation may promote the transformation of Aβ from a plaque state into a dissolved state, since the AS group showed higher soluble Aβ levels and lower insoluble Aβ levels than the SD group; acoustic stimulation may also accelerate tau protein clearance, since the AS group expressed lower total tau and phosphor-tau levels than the SD group. These results suggest that acoustic stimulation may reverse Aβ pathology and reduce tau protein levels exacerbated by chronic sleep deprivation.

In this study, we did not include a group containing mice that received acoustic stimulation and had normal sleep patterns, since a previous study [20] has already illustrated the beneficial impact of acoustic stimulation on AD mouse models without sleep deprivation. Apart from acting as a control group to verify that chronic sleep deprivation indeed worsened cognitive abilities and pathology in AD mouse models (through inter-group comparisons between the S and SD groups), the S group in our study also acted as a benchmark to measure the benefit of acoustic stimulation (through inter-group comparisons between the S and AS groups). We found that, although most indexes showed slight differences between the S and AS groups, such differences were not statistically significant. Therefore, we may assume that acoustic stimulation improves memory and reverses pathology in 3xTgAD mice suffering from chronic sleep deprivation, mimicking the effects of normal sleep patterns.

The significance of our study is that it demonstrates a protective effect of acoustic stimulation on AD mouse models, providing concrete evidence for the clinical use of acoustic stimulation; this treatment should be used at least in AD patients with chronic insomnia.

However, there are some limitations of our study. First, because of the lack of electroencephalography (EEG)/electromyography (EMG) recordings, we could not acquire quantitative data on sleep/wake patterns, meaning that we could not explore whether acoustic stimulation can stabilize and prolong NREM sleep as some clinical studies have suggested [15]. Second, although they showed typical cognitive decline and pathological changes, 8-month-old 3xTgAD mice may be too young to accurately represent elderly AD patients. Third, the absence of certain control groups (i.e., a group with a normal sleep pattern plus acoustic stimulation and a group of wild-type mice) may be a major limitation of the present study.

## 5. Conclusions

In conclusion, our study revealed that acoustic stimulation may exert a protective role in 3xTgAD mice, reversing the ill effects of chronic sleep deprivation on spatial memory, brain blood supply, Aβ pathology and the levels of tau proteins.

## Figures and Tables

**Table 1:** Skin lesions during chronic sleep intervention.

	Paw Lesions			Tail Lesions		
	S	SD	AS	S	SD	AS
Before	0	0	0	0	0	0
Middle	0	0	1	0	1	1
After	0	2	1	0	2	2

## Data Availability

The raw data supporting the conclusions of this article will be made available by the authors, without undue reservation.

## References

[1] Palmer R.F., Royall D.R. (2015). Future Dementia Severity is Almost Entirely Explained by the Latent Variable δ’s Intercept and Slope. J. Alzheimer’s Dis..

[2] Brookmeyer R., Johnson E., Ziegler-Graham K., Arrighi H.M. (2007). Forecasting the global burden of Alzheimer’s disease. Alzheimers Dement.

[3] Breijyeh Z., Karaman R. (2020). Comprehensive Review on Alzheimer’s Disease: Causes and Treatment. Molecules.

[4] Abeysinghe A., Deshapriya R., Udawatte C. (2020). Alzheimer’s disease; a review of the pathophysiological basis and therapeutic interventions. Life Sci..

[5] Mander B.A., Winer J.R., Walker M.P. (2017). Sleep and Human Aging. Neuron.

[6] Bubu O.M., Brannick M., Mortimer J., Umasabor-Bubu O., Sebastião Y.V., Wen Y., Schwartz S., Borenstein A.R., Wu Y., Morgan D. (2017). Sleep, Cognitive impairment, and Alzheimer’s disease: A Systematic Review and Meta-Analysis. Sleep.

[7] Cordone S., Annarumma L., Rossini P.M., De Gennaro L. (2019). Sleep and β-Amyloid Deposition in Alzheimer Disease: Insights on Mechanisms and Possible Innovative Treatments. Front Pharmacol..

[8] American Academy of Sleep Medicine (2007). Review articles for the AASM manual for the scoring of sleep and associated events: Rules, terminology, and technical specification. J. Clin. Sleep Med..

[9] Mander B.A., Rao V., Lu B., Saletin J.M., Lindquist J.R., Ancoli-Israel S., Jagust W., Walker M.P. (2013). Prefrontal atrophy, disrupted NREM slow waves and impaired hippocampal-dependent memory in aging. Nat. Neurosci..

[10] Diekelmann S., Born J. (2010). The memory function of sleep. Nat. Rev. Neurosci..

[11] Girardeau G., Lopes-Dos-Santos V. (2021). Brain neural patterns and the memory function of sleep. Science.

[12] Massimini M., Ferrarelli F., Esser S.K., Riedner B.A., Huber R., Murphy M., Peterson M.J., Tononi G. (2007). Triggering sleep slow waves by transcranial magnetic stimulation. Proc. Natl. Acad. Sci. USA.

[13] Marshall L., Molle M., Hallschmid M., Born J. (2004). Transcranial direct current stimulation during sleep improves declarative memory. J. Neurosci..

[14] Diep C., Ftouni S., Manousakis J.E., Nicholas C.L., Drummond S., Anderson C. (2020). Acoustic slow wave sleep enhancement via a novel, automated device improves executive function in middle-aged men. Sleep.

[15] Wunderlin M., Zust M.A., Hertenstein E., Feher K.D., Schneider C.L., Kloppel S., Nissen C. (2021). Modulating overnight memory consolidation by acoustic stimulation during slow-wave sleep: A systematic review and meta-analysis. Sleep.

[16] Ngo H.V., Martinetz T., Born J., Molle M. (2013). Auditory closed-loop stimulation of the sleep slow oscillation enhances memory. Neuron.

[17] Papalambros N.A., Santostasi G., Malkani R.G., Braun R., Weintraub S., Paller K.A., Zee P.C. (2017). Acoustic Enhancement of Sleep Slow Oscillations and Concomitant Memory Improvement in Older Adults. Front. Hum. Neurosci..

[18] Weigenand A., Molle M., Werner F., Martinetz T., Marshall L. (2016). Timing matters: Open-loop stimulation does not improve overnight consolidation of word pairs in humans. Eur. J. Neurosci..

[19] Schneider J., Lewis P.A., Koester D., Born J., Ngo H.V. (2020). Susceptibility to auditory closed-loop stimulation of sleep slow oscillations changes with age. Sleep.

[20] Martorell A.J., Paulson A.L., Suk H.J., Abdurrob F., Drummond G.T., Guan W., Young J.Z., Kim D.N., Kritskiy O., Barker S.J. (2019). Multi-sensory Gamma Stimulation Ameliorates Alzheimer’s-Associated Pathology and Improves Cognition. Cell.

[21] Iaccarino H.F., Singer A.C., Martorell A.J., Rudenko A., Gao F., Gillingham T.Z., Mathys H., Seo J., Kritskiy O., Abdurrob F. (2016). Gamma frequency entrainment attenuates amyloid load and modifies microglia. Nature.

[22] Ramanathan L., Siegel J.M. (2011). Sleep deprivation under sustained hypoxia protects against oxidative stress. Free Radic. Biol. Med..

[23] Everson C.A. (1995). Functional consequences of sustained sleep deprivation in the rat. Behav. Brain Res..

[24] Bergmann B.M., Kushida C.A., Everson C.A., Gilliland M.A., Obermeyer W., Rechtschaffen A. (1989). Sleep deprivation in the rat: II. Methodology. Sleep.

[25] Kushida C.A., Everson C.A., Suthipinittharm P., Sloan J., Soltani K., Bartnicke B., Bergmann B.M., Rechtschaffen A. (1989). Sleep deprivation in the rat: VI. Skin changes. Sleep.

[26] Vorhees C.V., Williams M.T. (2006). Morris water maze: Procedures for assessing spatial and related forms of learning and memory. Nat. Protoc..

[27] Sahu B., Mackos A.R., Floden A.M., Wold L.E., Combs C.K. (2021). Particulate Matter Exposure Exacerbates Amyloid-beta Plaque Deposition and Gliosis in APP/PS1 Mice. J. Alzheimers Dis..

[28] Gold C.A., Budson A.E. (2008). Memory loss in Alzheimer’s disease: Implications for development of therapeutics. Expert. Rev. Neurother..

[29] Solis E., Hascup K.N., Hascup E.R. (2020). Alzheimer’s Disease: The Link Between Amyloid-beta and Neurovascular Dysfunction. J. Alzheimers Dis..

[30] Bracko O., Cruz H.J., Park L., Nishimura N., Schaffer C.B. (2021). Causes and consequences of baseline cerebral blood flow reductions in Alzheimer’s disease. J. Cereb. Blood Flow. Metab..

[31] Hane F.T., Lee B.Y., Leonenko Z. (2017). Recent Progress in Alzheimer’s Disease Research, Part 1: Pathology. J. Alzheimers Dis..

